# Dentists’ opinions on knowledge, attitudes and barriers in providing oral health care to older people living independently in the Netherlands and Flanders (Belgium)

**DOI:** 10.1038/bdjopen.2017.20

**Published:** 2017-12-22

**Authors:** P C Bots-VantSpijker, J J M Bruers, C P Bots, L M J De Visschere, J M G A Schols

**Affiliations:** 1 BENECOMO, Flemish-Netherlands Geriatric Oral Research Group, Belgium, The Netherlands; 2 Department of Social Dentistry and Behavioral Sciences, Academic Centre for Dentistry Amsterdam (ACTA), University of Amsterdam and Vrije Universiteit, Amsterdam, The Netherlands; 3 Department of Research, Royal Dutch Dental Association (KNMT), Utrecht, The Netherlands; 4 Department of Oral Biochemistry, Academic Centre for Dentistry Amsterdam (ACTA), University of Amsterdam and Vrije Universiteit, Amsterdam, The Netherlands; 5 University Ghent, Faculty of Medicine and Health Sciences, Department of Dentistry, Community Dentistry and Oral Public Health Ghent, Belgium, The Netherlands; 6 Caphri/Department of Family Medicine and Department Health Service Research, Maastricht University, Maastricht, The Netherlands

## Abstract

**Objectives:**

The aim of this study was to investigate how dentists in the Netherlands and Flanders assessed their knowledge on oral health care to older people, what their attitude was and what barriers they experienced in rendering care to older people.

**Methods:**

The survey data was collected from a random sample of Dutch and Flemish dentists. Five hundred ninety-five dentists (37%) of the Dutch sample and 494 dentists of the Flemish sample (41%) completed the online questionnaire. Dentists were asked to respond to 15 Likert type items, representing opinions on provision of oral health care to older people and to give information about the number of older patients treated and about some profession-specific and personal characteristics.

**Results:**

The average number of patients treated per week was nearly twice as high in the Netherlands as in Flanders. Nevertheless, differences of opinions between dentists in the Netherlands and Flanders were relatively limited.

**Conclusions:**

This survey shows that in particular the actual number of older patients treated appears to be related with differences of opinions between Dutch and Flemish dentists about oral health care provided to (vulnerable) older people who live at home.

## Introduction

In many western countries, the proportion of older people within the population is on the increase and it is to be expected that this tendency will further continue in the years to come.^1^ It is known that becoming older is linked with a higher incidence of illnesses such as heart and vascular disease, dementia, diabetes mellitus, orthopaedic problems and neurological disease.^2^ These illnesses may lead to care dependency. With high care dependency, problems in maintaining good oral health can also be expected.^3^ Knowledge on oral health care to vulnerable older people is mainly based on research conducted amongst residents of nursing homes. However, not much is known about oral health care provided to older people who live at home independently. In general, it can be said that older people make less use of professional oral health care than younger people do. Factors such as illness, being less mobile and accordingly being housebound, insufficient financial means, fear and a low self-estimated demand for care might be related.^4–7^

Apart from the fact that older people may experience barriers in going to the dentist, dentists can also experience barriers to render oral health care to the elderly. Among other things this also becomes apparent from a survey by Bots-VantSpijker *et al.*^8^, conducted among dentists in the Netherlands. In line with this, it is interesting to investigate what the situation is in Flanders (Belgium). As for language, the level of prosperity and living conditions the region of Flanders in the neighbouring country of Belgium can be compared with the Netherlands. But distinctive differences between the two can also be reported. The number of inhabitants in the Netherlands is 16.8 millions of people, Flanders has 6.4 million inhabitants.^9^ Among other things, it appears that in Flanders there are relatively more practicing dentists. On the other hand, in the Netherlands, also oral hygienists are involved in providing professional oral health care. So, in the Netherlands oral health care is increasingly rendered in larger practices, in which various oral health care providers are collaborating.^9,10^ Furthermore, there are differences with regard to oral health. The DMFT index, indicating the extent of oral decay through caries, is lower in older people of 65 and over in Flanders than it is in the Netherlands.^9,11–14^ Also, the financing of oral health care for older people who live at home is different in both countries. In the Netherlands, in principle, these patients have to pay for the cost of oral health care themselves, although there are reimbursement possibilities depending on specific needs of care. In Flanders, it goes that patients will be reimbursed in all cases to a minimum of 75% of the cost.^9^

The aim of this study was to record how dentists in the Netherlands and Flanders assess their knowledge on oral health care to vulnerable older people, what their attitude is in this respect and what barriers they experience in rendering oral health care to these older people. The second aim was to consider whether there differences exist in these aspects between dentists in the Netherlands and in Flanders. For readability, Flanders will be mentioned as a country although it is in fact the Dutch speaking part of Belgium.

## Material and methods

### Data collection

The data were collected from a random sample of dentists in the Netherlands and Flanders by means of a web survey. In the Netherlands and Belgium, for survey research not involving patients, no ethical committee approval is required.

In December 2010, a sample of 1592 of the ~8,000 dentists in the Netherlands, aged 64 years or younger, were invited to participate in this study. The data collection was described earlier in the article of Bots-VantSpijker *et al.*^8^ In September 2011, a sample of 1,200 dentists In Flanders (26% of the total population of Flemish dentists) were invited to take part in the survey. These dentists were randomly selected out of the 3,500 members of the Verbond van Vlaamse Tandartsen (VVT), a dental association representing 76% of all practising 4,615 Flemish dentists.

The questionnaire, tested in a pilot by a dozen dentists, consisted of three parts. In the first part of the questionnaire some profession-specific and personal characteristics were recorded. In the second part, dentists were asked to give some information about the number of older patients they treated who still live at home. An inventory was made of how many patients per week they treat in their practice, how many of these are 75 years old or over and what percentage of these older patients they consider to be vulnerable. Vulnerable people were defined as people who experienced problems in physical, psychological and social areas (including financial problems). The age limit of 75 years was chosen, because it appeared that vulnerability increasingly occurs from that age.^14^

In part three of the questionnaire, dentists were asked to respond to 15 Likert type items representing opinions on the provision of oral health care to vulnerable older people who still live at home independently.

The web based questionnaire drawn up for this study was randomly sent by e-mail to the dentists in the samples. By using a personalized login and password, which was communicated in the e-mail, dentists obtained access to the online questionnaire via a secure website. In this way, confidential data collection was guaranteed. Dentists consented to participate in this study by sending their answers to the questions. All the dentists, in the Netherlands as well as in Flanders, who had not responded within two weeks and within five weeks consecutively, were sent a reminder by e-mail. The online survey period terminated six weeks after the start of the survey. Both the distribution of the questionnaires and the data management of the returned questionnaires were done by a research institute, independently from the authors, to ensure confidentiality.

### Statistical analysis

Since there were no differences between both study groups as regards to the questions asked and the data collection, the data from both countries was combined in a database and used for further statistical analysis.

After the analysis of some general and profession-specific characteristics of the dentists questioned, the division between the numbers of vulnerable older people that dentists said themselves they were treating was looked into. In doing so, the estimated percentage of vulnerable persons among the older patients who were treated was also determined and based on the distribution of these estimated percentages divided into quartiles. Subsequently, it was studied what the opinions of dentists in both countries are with regard to oral health care for these specific groups of patients and whether or not dentists in the Netherlands and Flanders differ from each other in this respect. Finally, it was assessed whether dentists who treat comparatively many (vulnerable) older people who live at home hold different views from dentists who proportionally treat fewer of these patients. In doing so, not only the differences within the Netherlands and Flanders were studied, but also the differences between the two countries. In these comparisons, the extremities were chosen as the starting points, i.e. only the dentists in the first and the fourth quartiles, respectively, were compared to each other. In this way, it was possible to show more clearly any correlation between treating relatively more or relatively less vulnerable older people and the forming of opinions. For these ‘first and fourth quartile’ dentists also the percentage of female dentists, the average age of dentists, the mean number of patients treated per week, and the mean number of patients treated per week aged 75 and over were also determined. By means of ANOVA and the Mann Whitney U test for ordinal data it was assessed whether the outcome of the ‘first and fourth quartile’ dentists showed any significant differences. The analyses were done using SPSS for Windows (Version 15, IBM SPSS, Armonk, NY, USA).

### Construction delta scores

The data representing the dentists’ opinions on the provision of oral health care to older people who still live at home showed no correlation from a statistical point of view. Therefore, the construction of one or more sum scores, based on the reactions of the dentists questioned on the 15 Likert type items, was not possible. Given the vast number of results accumulated by presenting these items separately, the data was subsequently processed in order to realise some data reduction. To this purpose, the Likert type items were first re-coded from five into three possible answers; (very much) agree received score 1, neutral score 0 and (very much) disagree score -1. This procedure resulted in the loss of some information, but it did make it possible to express the opinion of the dentists in one figure. The mean value per item of this so-called delta score (Δ) was between −100 and +100 and represented the difference between the proportion of dentists who ‘agree’ minus the proportion of dentists who ‘disagree’. Here it holds that the closer the value is to +1,00 the more positive the dentists were inclined towards the item concerned. But the various scores could only be interpreted relatively. A mean delta (Δ) of 0.80 is more positive than a mean delta of 0.55 but this difference is not to be interpreted as an absolute difference of 0.25 because of the specific processing. Therefore, the delta score has an ordinal character, not assuming the magnitude of difference between each category, but representing the tendency to agree or disagree upon an opinion. In this way delta scores were determined for all the Likert type items.

## Results

### General characteristics

In the end, 595 dentists (37%) of the Dutch sample completed the questionnaire. In Flanders 494 dentists (41%) did the same. As for gender, age, geographical location, graduation year and the university of qualification the total group of respondents proved to be fairly representative for the population of dentists in the Netherlands and Flanders.^8^

In Flanders, relatively more participating dentists were women, whereas the average number of patients treated per week was nearly twice as high in the Netherlands as in Flanders (Table 1).

### Situation per country

Furthermore, Table 2 shows that dentists in Flanders had in their practices a significantly greater proportion of vulnerable older people who live at home independently (22.2% vs 18.8%).

From the delta scores (Δ) presented in Table 3 it becomes clear that dentists in the Netherlands and Flanders only slightly differ in the way they look upon oral health care for vulnerable older people who live at home. In general, it can be concluded that reaction from Flemish dentists were slightly more positive with regard to treating vulnerable older people than those from their Dutch counterparts.

In the Netherlands, dentists are more confident that they have sufficient knowledge of the (adverse) effects of medication used by older people (0.42 vs 0.30). Flemish dentists more often believe that dental schools should pay more attention to provide students with adequate knowledge and skills with respect to the provision of oral health care to vulnerable older people (0.68 vs 0.60). They expressed more willingness to visit frail housebound older people for a regular dental check-up than dentists in the Netherlands (0.08 vs −0.08). Dutch dentists deny more often that treating vulnerable older people is not very challenging (−0.36 vs −0.27). But the dentist in Flanders sees fewer technical barriers compared to the dentist in the Netherlands. (0.52 vs 0.68). Dutch dentists deny more often that poor reimbursement for oral health care provisions to vulnerable older people forms a barrier to professional dedication towards this special patient group (−0.41 vs −0.27).

### The Netherlands versus Flanders

Table 4 shows the opinions on oral health care to vulnerable older people, distinguished into dentists in the Netherlands (group I) and Flanders (group II) who treat relatively few vulnerable older people and dentists who treat relatively many vulnerable older people (group III the Netherlands, group IV Flanders). It appears that in Flanders, the differences between these groups of dentists cover a somewhat wider range of opinions than in the Netherlands. In Flanders, as in the Netherlands, groups III and IV stated more often than groups I and II that they are capable of providing oral health care to cognitively impaired vulnerable older people and that they have experienced several times over that frail older people stopped coming to their clinics regularly. In addition, in Flanders, group IV showed more often than group II willingness to visit housebound frail older people for a regular dental check-up and stated more frequently that their practice is easily accessible to vulnerable older people and less frequently that providing oral health care to vulnerable older people is difficult due to its complexity and practical barriers.

In the comparison between Dutch and Flemish dentists who treat relatively few vulnerable older people (groups I and II), it is striking that they only differ significantly in one specific statement: The dentists in Flanders more often than the dentists in the Netherlands agree with the statement that daily attention for oral hygiene is a prerequisite for preventing oral health problems in dentate vulnerable older people.

In the comparison between dentists in the Netherlands and Flanders who treat relatively many vulnerable older people (groups III and IV), it is striking that Flemish dentists are more often prepared to visit housebound frail older people than Dutch dentists. In addition, they see fewer technical barriers. The dentists in the Netherlands who treat relatively many vulnerable older people indicate that to them, reimbursement forms a barrier less frequently than it does to dentists from Flanders. They also more often than their Flemish counterparts, endorse the statement that they have sufficient knowledge of the (adverse) effects of medication used by older people.

## Discussion and conclusions

From this study, it appears that there exist some differences of opinions between Dutch and Flemish dentists about oral health care provided to (vulnerable) older people who live at home on the subjects of knowledge, attitude and barriers. Table 5 offers a summary of the results.

### Interpretation of differences per country

It is remarkable that the Dutch dentists state more frequently that they have sufficient knowledge about the (adverse) effects of medication used by older people. Accordingly, the Flemish dentists stated more often that dental schools should pay more attention to furnishing students with adequate knowledge and skills with respect to providing oral health care to vulnerable older people. These differences may be explained by a diverse interpretation of the competences in geriatric dentistry in the Netherlands and Flanders.

The fact that the Flemish dentists saw fewer technical barriers in the rendering of oral health care to the elderly than their Dutch colleagues did, is in accordance with the fact that they were more prepared to make house calls. Based on clinical experience, this could have something to do with the smaller sized practices in Flanders: the smaller the practice, the more time to call on patients at home. It is also conceivable that in group practices there is more opportunity to free up time for making home visits. It is not clear how this difference could be explained. From a survey among German GPs about their attitude towards house calls, the main objection against making house calls was the reimbursement it yielded. Perhaps this argument also plays a part in the difference between the two countries in this study.^16^ In the Netherlands, the cost of oral health care are in more cases entirely for the patient himself, although it is possible to take out additional insurance. In Flanders, older people will get a 75% reimbursement for oral health care. Nevertheless, the Flemish dentists were more often convinced that inadequate financing forms a barrier in providing oral health care to vulnerable older patients. This indicates that differences in the financing of the dental services effect the providing of oral health care to frail older people. This is in accordance with the study of Holm-Pedersen *et al.*, who described that the availability of dental services, the organization of the dental health care delivery system, and price subsidy for dental treatment are significant mitigating factors that may influence the use of dental services among older people.^17^ Bots-VantSpijker *et al.*^8^ already demonstrated that the financial reimbursement was one of the barriers, and in addition some factors related to attitude and knowledge. Besides, the funding of oral health care in the Netherlands and Flanders is quite complex and the dentists in this study were not asked about this topic.

That dentists in Flanders more often than the dentists in the Netherlands agreed with the statement that daily attention for oral hygiene is a prerequisite for preventing oral health problems in dentate vulnerable older people, might be caused by the fact that the DMFT of people, aged 65–75 years old is lower in Flanders than in the Netherlands.^11,12^

### Interpretation of differences regarding number vulnerable older patients treated

In the comparison between dentists who treat relatively few and dentists who treat relatively many vulnerable older people, it comes to the latter felt more capable to do so. This can be explained and is in accordance with the findings of Ettinger,^18^ who described the importance of students spending sufficient clinical time with frail and medically compromised patients. Having sufficient clinical time, students may learn to develop rational treatment planning, and perform sufficient treatment procedures in order to feel clinically confident and comfortable towards the treatment of vulnerable and medically compromised patients. Although this study was conducted among students it is to be expected that the more vulnerable older patients one treats, the more capable one feels and will therefore also notice more often that patients no longer come to the practice. It is also remarkable, for that matter, that in the comparison between dentists who treat relatively few and relatively many vulnerable older patients, reimbursement did not appear to play a significant role, neither in the Netherlands, nor in Flanders.

### Limitations of the study

The response rate of the Dutch dentists (37%) and of the Flemish dentists (41%) is, compared to other web-based surveys, satisfactory.^19,20^ The number of dentists in this study is in accordance with 7.5% of the total number of dentists in the Netherlands and 10.7% of the total number of dentists in Flanders. In addition, it holds that the Dutch dentists involved in this study are representative of the population of dentists in that country, be it with a slight over representation of older dentists. A number of the Dutch dentists in this study belong to a group who regularly participate in surveys.^21^ This may have resulted in a positive bias, because these dentists are maybe motivated above average. The group of dentists from Flanders, all member of at least one organisation, has been approached for this study once only, resulting in a possibly lesser positive bias. In addition, it does not seem plausible that the timeline difference between both researches has influenced the outcome of the questionnaire.

Another limitation of this study is that the participants estimated the average numbers of patients treated per week aged 75 years or over, and those of vulnerable patients aged 75 years or over. It remains uncertain whether these estimations reflect the reality. To identify the exact average numbers of patients treated per week, dentists should have gathered information from their patient administrations. It seems unlikely that every dentist in this study did so.

In addition, the vulnerability of the group of older patients had to be estimated by dentists. Given the nature of this survey study it was not possible to apply a known vulnerability index, such as from Tilburg Frailty Indicator.^15^

The hypothesis that the season, in which the questionnaire was administered potentially, might have influenced the estimated numbers of patients treated per week aged 75 or over could not be excluded. Furthermore, the terms ‘complexity’ and ‘practical barriers’ were open to free interpretation and not defined in detail. Therefore, the lack of a detailed explanation may also have influenced the results.

Although both in the Netherlands and Flanders Dutch language is spoken, it is conceivable that in the two countries dentists have differently interpreted some statements. For example, in the Netherlands the dental consultation (with or without treatment) is seen as a procedure, resulting from a charging system, which is based on procedures. The Flemish dentist has possibly regarded the description of treatment more as an actual preventive or curative procedure.

### Conclusion

The differences in opinions between dentists in the Netherlands and Flanders, also taking into account the number of vulnerable older people they themselves are treating in their practice, appear to be relatively limited. More additional qualitative research in the dental practice on mainly attitude and barriers experienced is desirable.^22^ This will allow for the differences ascertained in this study to be better circumscribed. In addition, more insight is needed in the actual demand for oral health care to vulnerable older people who still live at home independently. What are the oral health problems with which these older patients confront dentists in the general practice and what care do dentists offer to this specific group of patients? Oral health care to vulnerable older people in the dental practice can only be further improved if more scientific and professional knowledge comes available.

## Declarations

Ethics: In the Netherlands and Belgium, for survey research not involving patients, no ethical committee approval is required. They survey includedquestions to dentists about characteristics of the practice they work in, as well as about their professional opinion and behaviour on non-sensitive matters. On avoluntarily basis they could decide to respond or not to respond to the request to participate in the survey. Dentists consented to participate in this study by sending in their answers to the questions. The survey was carried out by an independent third party research institute (theInstitute for Applied Social Sciences (ITS), which was linked to the Radboud University in Nijmegen, the Netherlands (www.ru.nl/its/) until May 2016) commissioned by the Royal Dutch Dental Association (Koninklijke Nederlandse Maatschappijtot bevordering der Tandheelkunde, KNMT). The independent third party research institute confidentially collects and manages all research data in such a way that researchers cannot trace any of the data to anindividual dentist or practice. The institute carried out the distribution of the questionnaires, the data management of the returned questionnaires, the interlinking of the research data with basic information of dentists and the anonymization of the data (necessary to make the research database available for the actual researchers).

### Data availability

Commissioned by the Royal Dutch Dental Association (KNMT), the data are stored and managed by an independent research institute, which operates as a third party and is affiliated with the Radboud University Nijmegen. If others would like to use the data, a request must be made directly to KNMT to provide an anonymous version of the research database. The contact details of the third party are: KNMT: j.bruers@knmt.nl (research coordinator KNMT).

## Figures and Tables

**Table 1:** Personal and professional characteristics of dentists, per (part of the) country

	*The Netherlands*	*Flanders*	*Total*
Proportion female (in %)^a^	24	48	34^b^
Age in January 2011 (in %)^c^			*
39 or younger	20	19	20
40–54	44	52	47
55–65	36	29	33
Mean age (s.d.)	49.4 (9.9)	48.4 (10.2)	49 (10)
Year of dental graduation (in %)			
1979 or earlier	25	31	28
1980–1989	43	39	41
1990–1999	15	18	16
2000 or later	17	12	15
Mean year (s.d.)	1986.8 (10)	1985.7 (10)	1986.3 (10)
Proportion practice owners (in %)^a^	84	90	87^b^
Mean (sd) number of patients treated^d^	105.3 (58)	58.2 (24.8)	84.7 (52)^b^
Mean (sd) number of patients treated, aged 75 years and over^d^	8.0 (8.7)	6.3 (5.3)	7.2 (7.5)^b^
*N*	589–595	422–458	1.017–1.055

a Dummy variable (1/0).

b F-test: *P*<0.05.

c Chi Square test: *P*<0.05 / Cramer’s V<0.15.

d In the previous full working week in the practice by the dentist himself.

**Table 2:** Percentage of ‘vulnerable’ patients within the number of patients, aged 75 years and over, treated by the dentist in the previous full working week, per (part of the) country

	*The Netherlands*	*Flanders*	*Total*
0% of the patients (1st quartile)	24%	26%	25%
1–9% of the patients (2nd quartile)	27%	18%	23%
10–29% of the patients (3rd quartile)	24%	24%	24%
30–100% of the patients (4th quartile)	25%	32%	28%
Mean	18.8	22.2	20.3^a^
Median	8	10	10
Modus	0	0	0
s.d.	24.8	26.2	25.5
Maximum	100	100	100
Minimum	0	0	0
*N*	590	450	140

a F-test: *P*<05.

**Table 3:** Participants’ opinions on oral health care provision to vulnerable older people who live at home, per (part of the) country

	*The Netherlands (NL)*	*Flanders (FL)*	*Total*	P*-value*
	Δ (agree–disagree)	Δ (agree–disagree)	Δ (agree–disagree)	NL versus FL
K1	Physical, psychological, and social aspects have an impact on oral health care decision making			
	**0.92** (0.93–0.01)	**0.90** (0.92–0.02)	**0.91** (0.93–0.02)	0.55
K2	I have sufficient knowledge of the (adverse) effects of medication used by older people.			
	**0.42** (0.56–0.15)	**0.30** (0.52–0.21)	**0.37** (0.54–0.18)	**0.04**^a^
K3	I am capable of providing oral health care to cognitively impaired vulnerable older people.			
	**0.39**(0.53–0.14)	**0.46** (0.58–0.12)	**0.42** (0.55–0.13)	0.14
K4	Dental schools should pay more attention to providing students with adequate knowledge and skills with respect to oral health care provision to vulnerable older people.			
	**0.60** (0.67–0.07)	**0.68** (0.73–0.06)	**0.64** (0.70–0.06)	**0.04**^a^
K5	Daily attention for oral hygiene care is a prerequisite for preventing oral health problems in dentate vulnerable older people.			
	**0.95** (0.96–0.01)	**0.97** (0.97–0.01)	**0.96** (0.97–0.01)	0.24
A1	Every dentist is responsible for providing proper oral health care to housebound frail older people who used to visit his clinic regularly.			
	**0.27** (0.49–0.21)	**0.24** (0.47–0.23)	**0.26** (0.48–0.22)	0.48
A2	I am willing to visit housebound frail older people for a regular dental check-up.			
	**-0.08** (0.38–0.46)	**0.08** (0.44–0.37)	**-0.01** (0.40–0.42)	**0.01**^a^
A3	I have experienced several times over that, at a certain moment, (frail) older people stopped coming to the practice regularly.			
	**0.62** (0.73–0.11)	**0.70** (0.77–0.08)	**0.65** (0.75–0.10)	0.08
A4	From a dentist’s point of view, treating vulnerable older people is not very challenging.			
	**-0.36** (0.19–0.55)	**-0.27** (0.21–0.48)	**-0.32** (0.20–0.52)	**0.04**^a^
B1	Opportunities to refer vulnerable older people with complex oral health problems to a colleague with specific knowledge and skills are limited.			
	**0.67** (0.74–0.07)	**0.67** (0.75–0.08)	**0.67** (0.74–0.08)	0.98
B2	Providing oral health care to vulnerable older people is difficult due to its complexity and practical barriers.			
	**15.7** (45.5–29.8)	**22.0** (59.9–27.9)	**18.5** (47.4–29.0)	0.24
B3	The reimbursement of oral health care provision to vulnerable older people is poor.			
	**0.16** (0.46–0.30)	**0.22** (0.60–0.28)	**0.19** (0.47–0.29)	0.84
B4	My practice is easily accessible for vulnerable older people, without major obstacles.			
	**0.72** (0.81–0.10)	**0.71** (0.79–0.08)	**0.71** (0.80–0.09)	0.48
B5	Usually, oral health care for vulnerable older people implies restraints with regard to technical facilities.			
	**0.68** (0.76–0.08)	**0.52** (0.63–0.11)	**0.61** (0.70–0.09)	**0.00**^a^
B6	I regard the poor reimbursement of oral health care provision to vulnerable older people as a barrier to professional dedication to this special patient group.			
	**−0.41** (0.15–0.56)	**−0.27** (0.19–0.46)	**−0.35** (0.17–0.52)	**0.00**^a^
	*n*=517–553	*n*=403–425	*n*=920–978	

K1–K5—opinions on knowledge.

A1–A4—opinions on attitude.

B1–B6—opinions on barriers

Agree—proportion dentists who agree with an opinion.

Disagree—proportion dentists who disagree with an opinion.

Δ—mean difference score (Likert type items were recoded from five into three possible answers: (very much) agree (score 1) neutral (score 0) and (very much) disagree (score −1). Following this, a delta score (Δ) was calculated by taking the mean of the scores per statement, which represents the difference between proportion dentists who ‘agree’ minus proportion dentists who ‘disagree’).

a Mann-Whitney U-test: *P*<05.

**Table 4:** Dentist’ characteristics and professional attitudes on oral health care to vulnerable elderly, per (part of the) country

	*The Netherlands (NL)*		*Flanders (FL)*		*Comparisons*			
	*Less 1st quartile (groupI)*	*High 4th quartile (group III)*	*Less 1st quartile (group II)*	*More 4th quartile (group IV)*	P*-values NL-less versus NL-more (I–III)*	P*-values FL-less versus FL-more (II–IV)*	P*-values NL-less versus FL-less (I–II)*	P*-values NL-more versus FL-more (III–IV)*
	*NL-less*	*NL-more*	*FL-less*	*FL-more*				
Female dentists (in %)	32.6	24.7	61.8	49.6	*0.13*	*0.06*	***0.00***^a^	***0.00***^a^
Mean age of dentists	49.0	48.4	46.7	47.5	*0.62*	*0.55*	*0.09*	*0.42*
Mean number of patients treated per week	98.3	106.0	51.3	56.4	*0.30*	*0.07*	***0.00***^a^	***0.00***^a^
Mean number of patients treated per week, aged 75 years and over	2.6	8.8	3.0	6.5	***0.00***^a^	***0.00***^a^	*0.32*	***0.02***^a^
Δ K1	0.92	0.96	0.90	0.90	*0.21*	*0.89*	*0.86*	*0.11*
Δ K2	0.37	0.45	0.17	0.24	*0.31*	*0.51*	*0.07*	***0.03***^b^
Δ K3	0.18	0.41	0.28	0.50	***0.01***^b^	***0.02***^b^	*0.27*	*0.31*
Δ K4	0.70	0.63	0.69	0.71	*0.34*	*0.83*	*0.72*	*0.25*
Δ K5	0.92	0.96	0.98	0.96	*0.10*	*0.17*	***0.03***^b^	*0.46*
Δ A1	0.28	0.32	0.23	0.24	*0.66*	*0.99*	*0.70*	*0.37*
Δ A2	-0.17	-0.19	-0.07	0.23	*0.88*	***0.01***^b^	*0.37*	***0.00***^b^
Δ A3	0.51	0.71	0.57	0.73	***0.05***^b^	***0.04***^b^	*0.93*	*0.89*
Δ A4	-0.31	-0.41	-0.30	-0.28	*0.37*	*0.92*	*0.64*	*0.11*
Δ B1	0.68	0.75	0.69	0.70	*0.32*	*0.95*	*0.73*	*0.57*
Δ B2	0.26	0.17	0.35	0.13	*0.38*	***0.03***^b^	*0.34*	*0.67*
Δ B3	0.58	0.53	0.49	0.62	*0.85*	*0.18*	*0.51*	*0.34*
Δ B4	0.62	0.76	0.57	0.74	*0.10*	***0.01***^b^	*0.32*	*0.85*
Δ B5	0.64	0.71	0.54	0.60	*0.45*	*0.79*	*0.18*	***0.04***^b^
Δ B6	−0.32	-0.46	-0.40	-0.27	*0.12*	*0.17*	*0.47*	***0.02***^b^
Positive about guideline	0.79	0.77	0.77	0.85	*0.67*	*0.11*	*0.66*	*0.11*
*N*	*114–141*	*136–150*	*102–116*	*127–144*				

Δ—mean difference score (Likert type items were recoded from five into three possible answers: (very much) agree (score 1) neutral (score 0) and (very much) disagree (score −1). Following this, a delta score (Δ) was calculated by taking the mean of the scores per statement, which represents the difference between proportion dentists who ‘agree’ minus proportion dentists who ‘disagree’.).

K1–K5—opinions on knowledge.

K1—Physical, psychological, and social aspects have an impact on oral health care decision-making.

K2—I have sufficient knowledge of the (adverse) effects of medication used by older people.

K3—I am capable of providing oral health care to cognitively impaired frail older people.

K4—Dental schools should pay more attention to teaching students adequate knowledge and skills with respect to oral health care provision to vulnerable older people.

K5—Daily attention for oral hygiene is a prerequisite for preventing oral health problems in dentate vulnerable older people.

A1–A4 opinions on attitudes.

A1—Every dentist is responsible for providing proper oral health care to housebound frail older people who used to visit his clinic regularly.

A2—I am willing to visit housebound frail older people for a regular dental check-up.

A3—I have experienced several times over that, at a certain moment, (frail) older people stopped coming to the clinic regularly.

A4—From a dentist’s point of view, treating vulnerable older people is not very challenging.

B1–B6—opinions on barriers.

B1—Opportunities to refer vulnerable older people with complex oral health care problems to a colleague with specific knowledge and skills are limited.

B2—Providing oral health care to vulnerable older people is difficult due to its complexity and practical barriers.

B3—The reimbursement of oral health care provision to vulnerable older people is poor.

B4—My practice is easily accessible for vulnerable older people, without major obstacles.

B5—Usually, oral health care to vulnerable older people implies restraints with regard to technical facilities.

B6—Poor reimbursement of oral health care provision to vulnerable older people is a barrier for my professional dedication to this special patient group.

a *F*-test: *P*<0.05.

b Man–Whitney *U*-test: *P*<0.05.

**Table 5:** Summary of the results of Tables 3 and 4

*Statement*	*Table 3*	*Table 4*			
		*l–h Neth*	*l–h Fl*	*l-l Neth/Fl*	*h-h Neth/Fl*
K1	0	0	0	0	0
K2	−(NL>FL)	0	0	0	−(NL>FL)
K3	0	−(H>L)	−(H>L)	0	0
K4	+ (FL>NL)	0	0	0	0
K5	0	0	0	+ (FL>NL)	0
A1	0	0	0	0	0
A2	+ (FL>NL)	0	−(H>L)	0	+ (FL>NL)
A3	0	−(H>L)	−(H>L)	0	0
A4	+(FL>NL)	0	0	0	0
B1	0	0	0	0	0
B2	0	0	+ (L>H)	0	0
B3	0	0	0	0	0
B4	0	0	−(H>L)	0	0
B5	−(NL>FL)	0	0	0	−(NL>FL)
B6	+ (FL>NL)	0	0	0	+ (FL>NL)

Score 0, no significant difference between the Netherlands and Flanders or between low and high number of vulnerable elderly treated.

Score−, the outcome measure of Flanders is smaller than that of the Netherlands or the outcome measure for a low number of patients treated is smaller than for a high number of patients treated.

Score+, the outcome measure of Flanders is bigger than that of the Netherlands or the outcome measure for a low number of patients treated is bigger than for a high number of patients treated.

K1–K5—opinions on knowledge.

K1—Physical, psychological, and social aspects have an impact on oral health care decision-making.

K2—I have sufficient knowledge of the (adverse) effects of medication used by older people.

K3—I am capable of providing oral health care to cognitively impaired frail older people.

K4—Dental schools should pay more attention to teaching students adequate knowledge and skills with respect to oral health care provision to vulnerable older people.

K5—Daily attention for oral hygiene is a prerequisite for preventing oral health problems in dentate vulnerable older people.

A1–A4—opinions on attitudes

A1—Every dentist is responsible for providing proper oral health care to housebound frail older people who used to visit his clinic regularly.

A2—I am willing to visit housebound frail older people for a regular dental check-up.

A3—I have experienced several times over that, at a certain moment, (frail) older people stopped coming to the clinic regularly.

A4—From a dentist’s point of view, treating vulnerable older people is not very challenging.

B1–B6—opinions on barriers.

B1—Opportunities to refer vulnerable older people with complex oral health care problems to a colleague with specific knowledge and skills are limited.

B2—Providing oral health care to vulnerable older people is difficult due to its complexity and practical barriers.

B3–The reimbursement of oral health care provision to vulnerable older people is poor.

B4—My practice is easily accessible for vulnerable older people, without major obstacles.

B5—Usually, oral health care to vulnerable older people implies restraints with regard to technical facilities.

B6—Poor reimbursement of oral health care provision to vulnerable older people is a barrier for my professional dedication to this special patient group.
